# Supplementary material for: Ecological network analysis reveals cancer-dependent chaperone-client interaction structure and robustness
Source: Nat Commun. 2023 Oct 7;14:6277. doi: 10.1038/s41467-023-41906-2 (PMC10560210; doi:10.1038/s41467-023-41906-2)
Supplement: Supplementary file 1 — Supplementary Information [file 41467_2023_41906_MOESM1_ESM.pdf]

Ecological network analysis reveals cancer-dependent chaperone-client interaction structure and robustness

Geut Galai [1], Xie He [2], Barak Rotblat [3,1], and Shai Pilosof [1,*]

[1] *Department of Life Sciences, Ben-Gurion University of the Negev, Beer-Sheva, Israel*
[2] *Department of Mathematics, Dartmouth College, 27 N Main St, Hanover, NH 03755, USA.*
[3] *The National Institute for Biotechnology in the Negev, Beer Sheva 8410501, Israel.*
[*] *Corresponding author: pilos@bgu.ac.il*

Supplementary Materials

Table S1. Summary of results for normalizing the significant chaperone-client correlations by number of samples in each cancer.

Abbreviation	Cancer	Samples	All Correlations	N valid	% valid
KIRP	Kidney renal papillary cell carcinoma	288	5108	5108	100%
LIHC	Liver hepatocellular carcinoma	371	3693	2148	58.16%
STAD	Stomach adenocarcinoma	375	5015	3083	61.48%
COAD	Colon adenocarcinoma	478	5665	2840	50.13%
PRAD	Prostate adenocarcinoma	498	6236	3661	58.71%
HNSC	Head and Neck squamous cell carcinoma	500	7466	3612	48.38%
LUSC	Lung squamous cell carcinoma	502	4641	1463	31.52%
THCA	Thyroid carcinoma	502	6945	4305	61.99%
LUAD	Lung adenocarcinoma	533	4577	1522	33.25%
KIRC	Kidney renal clear cell carcinoma	538	6242	3089	49.49%
UCEC	Uterine Corpus Endometrial Carcinoma	551	6461	3201	49.54%
BRCA	Breast invasive carcinoma	1102	6358	1411	22.19%

For each cancer tissue we detail the number of samples in each cancer cohort, the number of coexpression correlations found to be significant using Spearman correlations, the number of correlations found to be valid after bootstrapping (see Methods), and the proportion of correlation kept after bootstrapping, out of the number of the significant correlations using Spearman alone.

Table S2. List of the chaperones used in this study.

Chaperone Name	Functionality	Co-chaperone
SPG7	Protease	No
CLPP	Protease	Yes
HSPE1	Folding	Yes
DNAJC19	Folding	Yes
HTRA2	Protease	No
HSCB	Folding	Yes
LONP1	Protease	Yes
AFG3L2	Protease	No
HSPA9	Folding	No
CLPX	Protease	No
HSPD1	Folding	No
DNAJA3	Folding	Yes
TRAP1	Folding	No
GRPEL2	Folding	Yes
YME1L1	Protease	No

Table S3. Spearman correlation values (two tailed) for the correlations of realized niche with chaperone expression for each chaperone.

**chaperone**	**p-value**	**r value**
AFG3L2	0.931	0.028
CLPP	0.753	0.102
CLPX	0.404	-0.266
DNAJA3	0.404	-0.266
DNAJC19	0.746	-0.105
GRPEL2	0.145	-0.448
HSCB	0.829	-0.0699
HSPA9	0.297	-0.329
HSPD1	0.527	0.203
HSPE1	0.255	-0.357
HTRA2	0.276	-0.343
LONP1	0.966	0.014
SPG7	0.131	0.462
TRAP1	0.379	0.280
YME1L1	0.00824	-0.720

The Bonferroni-corrected significant p-value is 0.003.

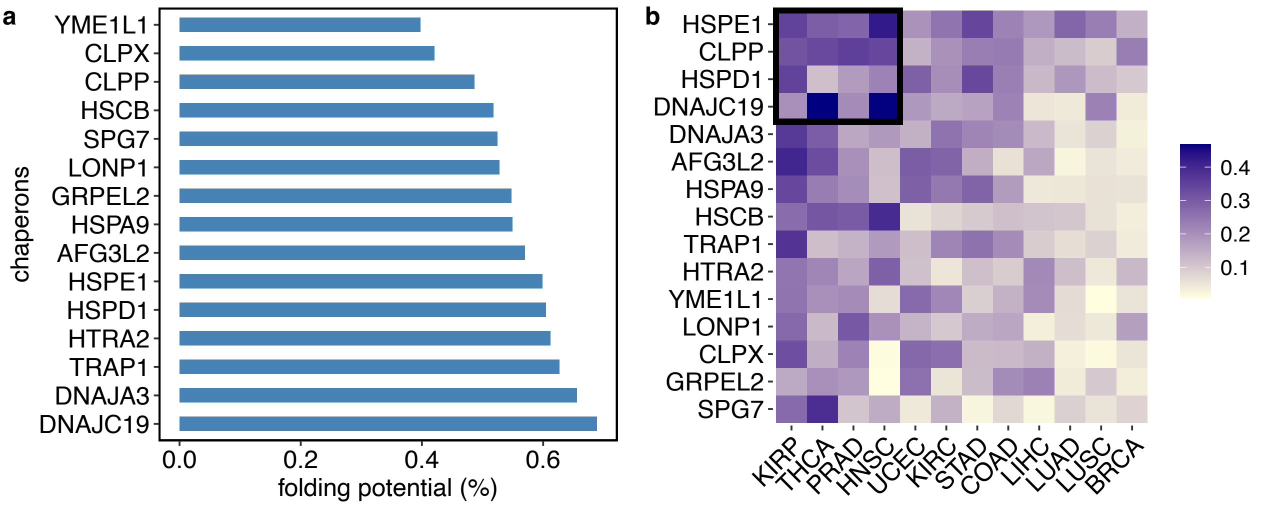

Figure S1. **chaperone specialization.** **a** Specialization S_c is a proportion calculated as the total number of clients that a chaperone interacts with across cancers, out of all the 1,142 mitochondrial proteins. **b** Each square in the heatmap depicts a chaperone's specialization in cancer α, $S_c{}^{\alpha}$, defined as the number of clients that a chaperone (rows) interacts with in a given cancer α (columns), $L^{\alpha}{}_{c}$, out of all the proteins ($S_c{}^{\alpha} = L^{\alpha}{}_{c}/1,142$; see Results). The non-uniform colors in each row indicate that each chaperone interacts with a different number of clients across cancers. The non-uniform colors in each column indicate that cancer types also vary in the extent to which they enable chaperones to interact with clients. Put together, these two observations create a weighted-nested pattern whereby the more specialized chaperones interact with less clients and are a subset of the more generalist ones; on the other hand, cancer environments that enable chaperones to interact with less clients are subsets of those that allow for higher levels of generalism. Weighted-nestedness was statistically significantly non-random when compared to 1,00 counterpart networks assembled from networks in which chaperone-client interactions were shuffled (Methods; Fig. S2A).Therefore, there is a non-randomly structured way in which cancer environments mediate the interactions of chaperones. Rows and columns are arranged by their marginal sums.

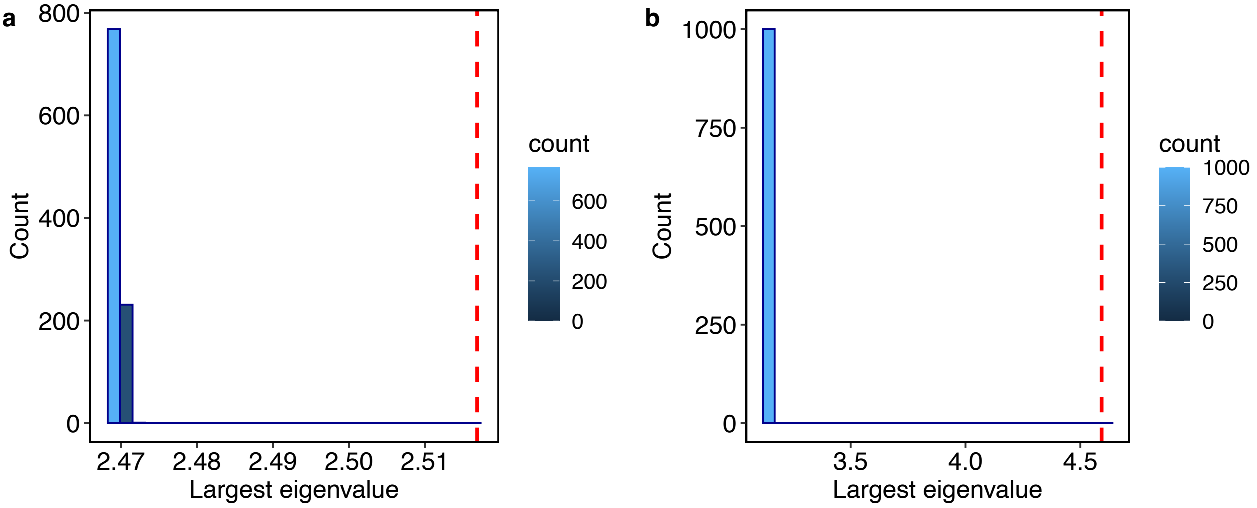

Figure S2. **Significance of weighted nestedness.** Nestedness was calculated as the largest eigenvalue of each matrix in Fig. 1 in the main text. This value was compared to a distribution of 1,000 values calculated for shuffled networks. The observed value (dashed vertical line was larger than all values obtained for shuffled networks for specialization **a** (n=1000) and realized niche **b** (n=1000).

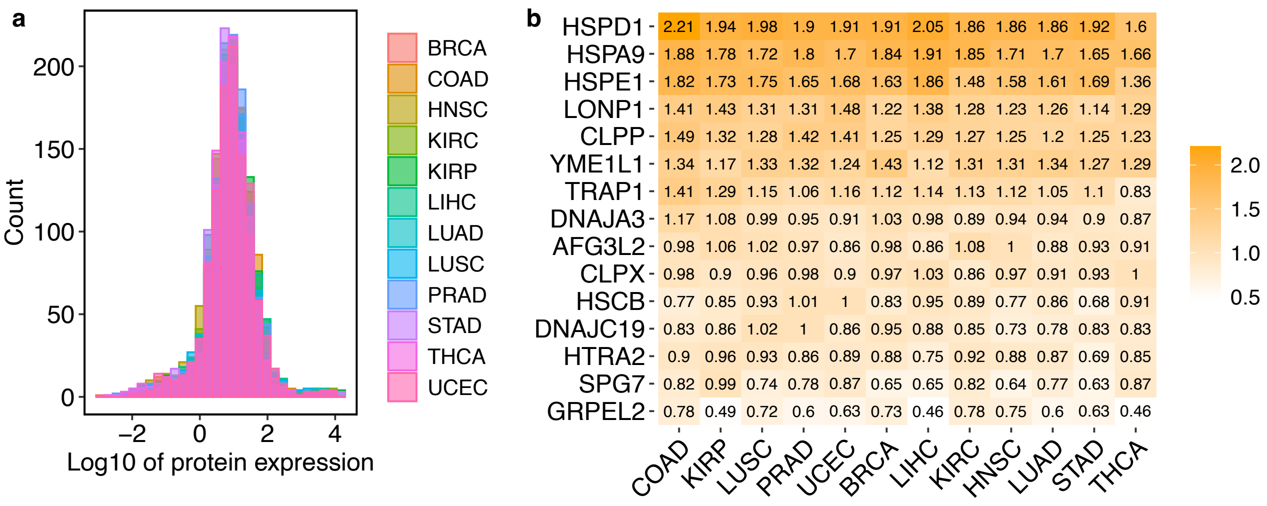

Figure S3. **Gene expression levels are similar across cancers but vary across chaperones.** **a** The median expression value of each mitochondrial protein was calculated and log-transformed for comparable expression scales. Each color depicts the distribution of these medians in a given cancer. **b** The median value for each chaperone's expression level in each cancer type was calculated, and transformed by log10 to compare expression scale. Each value is presented in the corresponding cell in the heatmap. Rows and columns are arranged by their marginal sums.

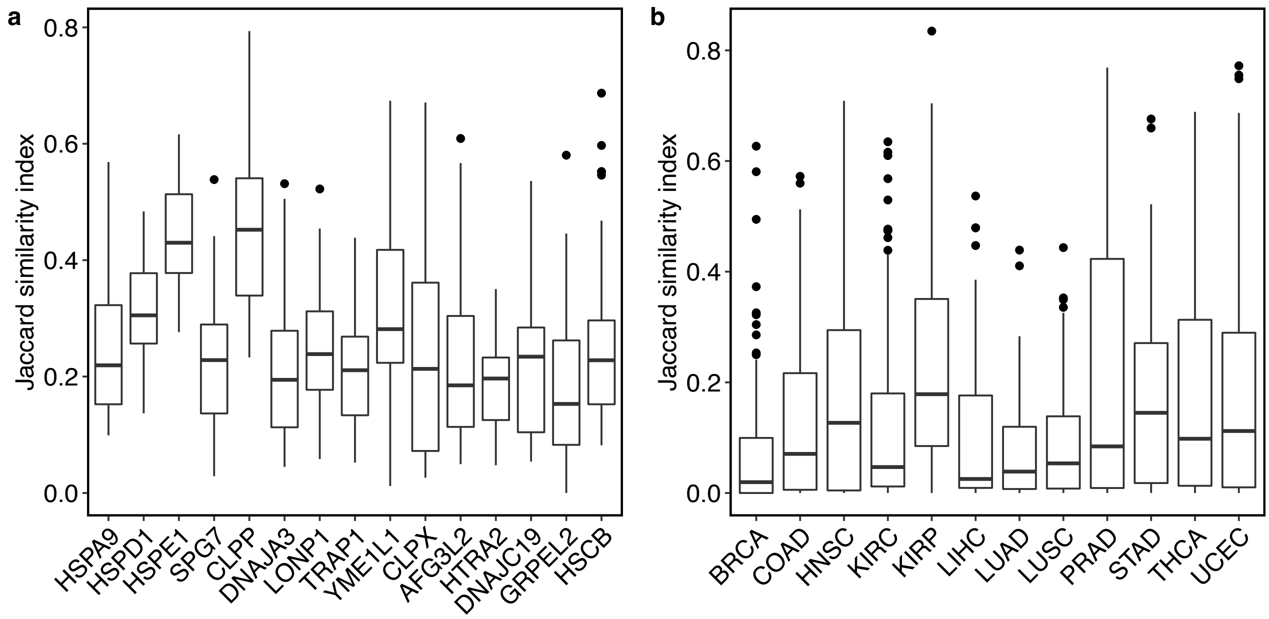

Figure S4. **Jaccard similarity index distributions.** **a** Distribution of Jaccard similarity for the identity of clients for each chaperone c between all pairs of cancer types α and β ($J_c{}^{\alpha\beta}$). **b** Distribution of Jaccard similarity for the identity of clients for every pair of chaperones x and y, within each cancer type α ($J_{xy}{}^{\alpha}$). Box plots: horizontal line is median, lower and upper hinges are the 25th and 75th percentiles, lower and upper whiskers are 1.5*IQR, points are outliers.

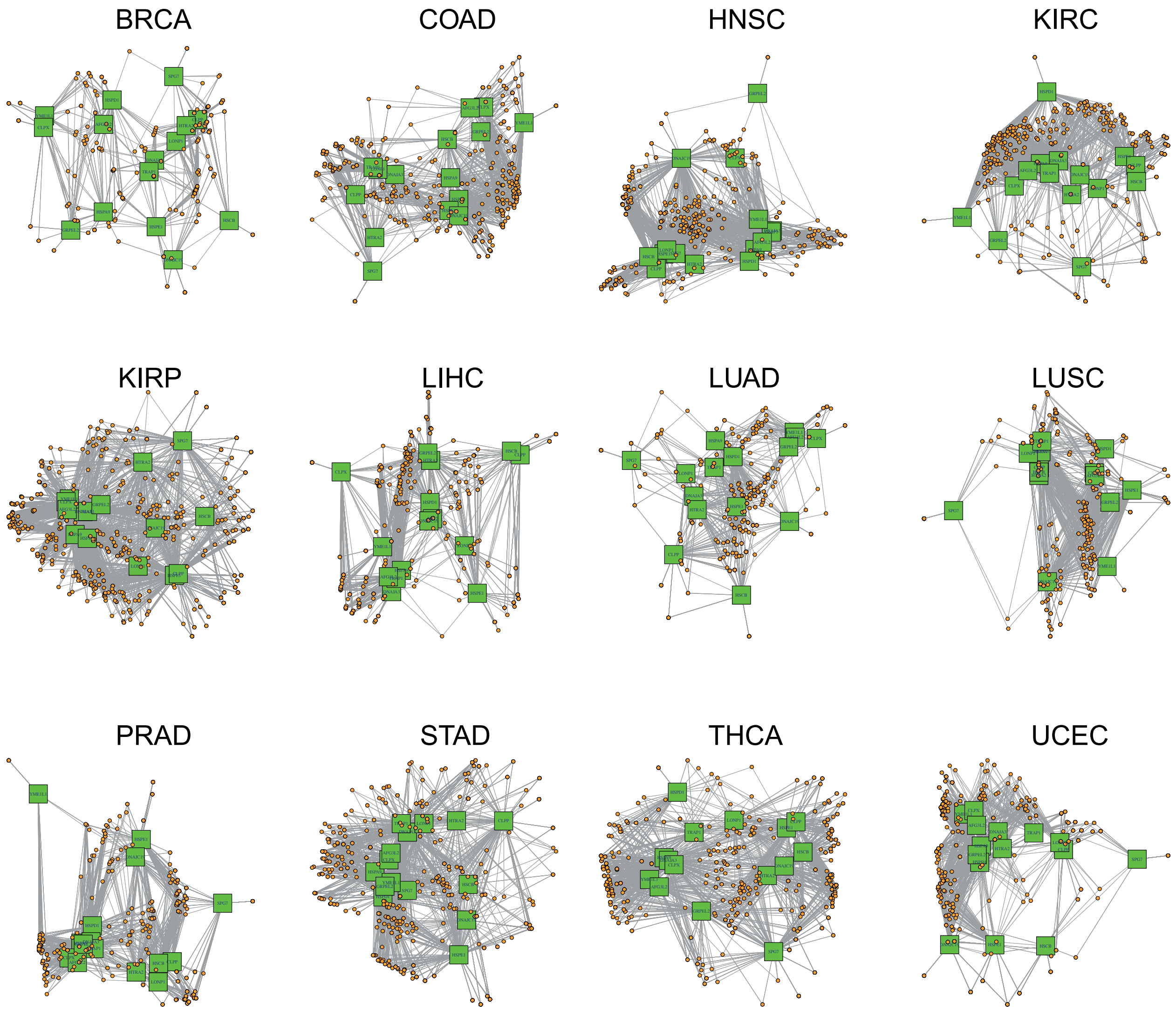

Figure S5. **Chaperon-client networks.** Chaperones and clients are depicted by green squares and orange circles, respectively. A larger version of each network image can be found on the Github repository.

Supplementary note 1: validating coexpression data

To predict the consequence of targeting a particular mitochondrial chaperone in a particular cancer type, we need to understand better which proteins depend upon which chaperones for their folding in a particular cancer type. The challenge is that we know little about which proteins collapse when targeting a particular mitochondrial chaperone. While a few examples are cited in the introduction, these important functional studies were performed using specific cell lines growing in culture. Therefore, they do not provide complete information regarding the chaperones' targets in human tumors. In particular, we do not know what are the substrates of a given chaperone are in different cancer types.

To overcome these limitations, we used mRNA expression data derived from human tumors in different cancer types. We assume that in cases where a mitochondrial chaperone is a limiting factor in the folding of a particular protein in a particular cancer type, there will be selection towards coexpression of the chaperone and its target in that tumor type [1]. The question is if coexpression, as reflected in mRNA levels of two genes, indicates protein-protein interactions and if mRNA expression data can predict protein-protein interaction networks in disease. A body of work shows this is indeed the case [1–4], and specifically, that coexpression predicts protein-complex stoichiometry in the OXPHOS system [5].

We further reinforced the support from the literature for using coexpression data as follows. We chose three chaperones for which experimental data on clients in the mitochondria is available: HSPD1 [6], CLPP [7] and TRAP1 [8,9]. We then calculated the proportion of coexpression interactions we found that were observed experimentally in these papers. Given that experimentally detecting interactions is a daunting task, we a-priori expected relatively low validation rates because: (i) These studies were preformed in particular human cell lines from a particular tumor type and, therefore, may differ from interactions occurring in tumors growing in vivo and in different tumor types. (ii) Even in empirical studies the definition for an interaction may not necessarily mean binding. Specifically in these studies 'interaction' refers to proteins aggregating upon down regulation or inhibition of a specific chaperone or, in the case of CLPP, proteins that are eliminated upon activation of the protease. While these are good indication for interaction, thy may have occurred for reasons which are not directly linked to chaperone-client binding.

Nevertheless, we found experimental evidence for 12%-29% of the coexpression interactions. While this rate of empirical evidence is high, we wanted to test it statistically. To show that this proportion of experimental validation is more significant than expected, we randomly sampled from the mitochondrial proteins a set of genes (number of genes sampled was equal to the number of proteins each chaperone had) 1,000 times. Each time we recorded how many interactions had experimental evidence. We found that the empirical evidence for coexpression interactions is statistically significantly greater than the baseline evidence (Fig. S6A).

While this experimental validation provided a starting point, it did not encompass all the chaperones. Doing so requires a standardized database that encompasses all experimental evidence. For each of our 15 chaperones, we obtained experimental interactions from the STRING database [10] with *Homo sapiens* genes in the experimental evidence channel. Here too we expected low validation rates for the reasons described above. Nevertheless, we found experimental evidence for 1%-31% of the coexpression interactions across chaperones (Fig. S6B). Here too it is imperative to show statistical support. In addition, the extent of experimental evidence largely depends on sampling effort because some chaperones are more studied than others. The question is whether this amount of empirical evidence is greater than what one would expect at random in studies of our kind. For each chaperone, we randomly sampled 10,000 times a set of genes from the whole human genome. The sampled set size was equal to the number of clients we discovered using coexpression. For each random set, we calculated the proportion of genes that has experimental evidence for an interaction with the chaperone. We found that the experimental evidence for the coexpression interactions was statistically significantly greater than the evidence for interactions with other genes. This was true

for all the chaperones besides SPG7 (Fig. S6B).

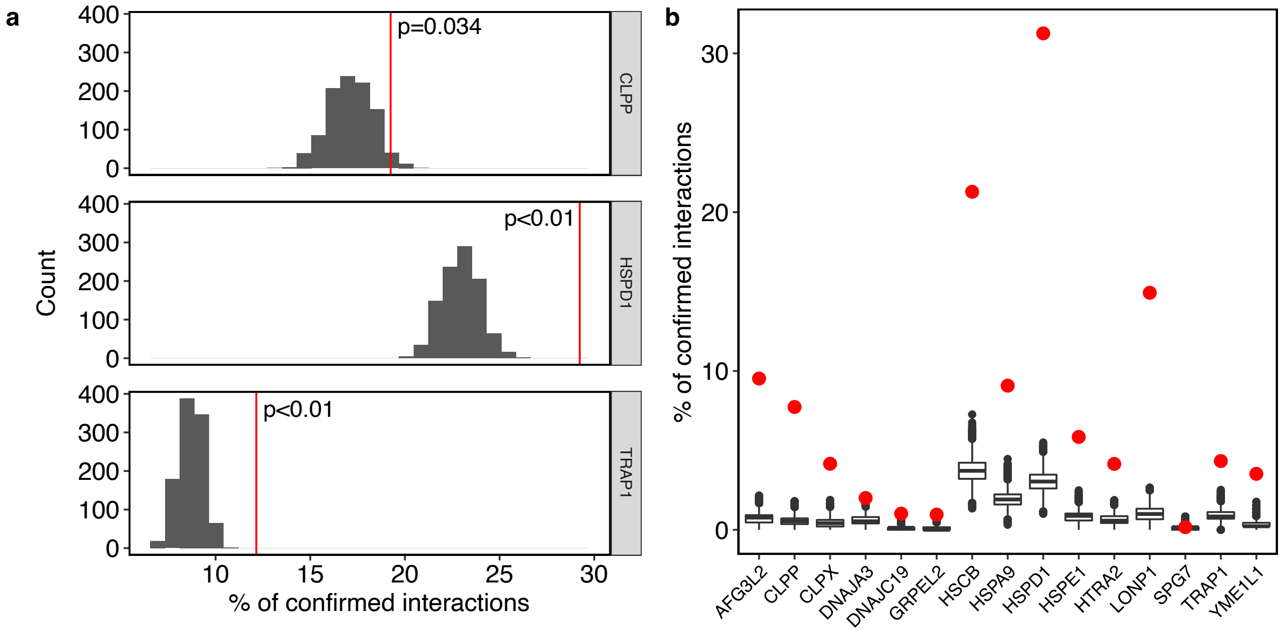

Figure S6. **Experimental evidence for chaperone-client interactions.** **a** Three chaperones for which a list of clients obtained experimentally was available. Vertical red lines depict the observed proportion of validated coexpression interactions. Histograms show the distribution of confirmed interactions with those chaperones found when randomly sampling from mitochondrial-client genes (n=1000). **b** The red point for each chaperone is the proportion of interactions discovered using coexpression that were empirically validated in the STRING database. The box plots show the distribution of confirmed interactions with the relevant chaperone found by randomly sampling genes (n=10,000) from the whole human genome. Boxplots: horizontal line is median, lower and upper hinges are the 25th and 75th percentiles, lower and upper whiskers are 1.5*IQR, points are outliers.

Supplementary note 2: community detection

In addition to the SBM we detected the optimal partitioning to modules using Infomap [11,12]. Briefly, Infomap is an unsupervised clustering algorithm, which detects an optimal network partition based on the movement of a random walker on the multilayer network (see [11–13] for details). For any given partition of the network, the random walker moves across nodes in proportion to the weight of the edges. Hence, it will tend to stay longer in dense areas. These areas can be defined as 'modules'. The movement dynamics can be converted to an information-theoretic currency using the objective function L called the map equation. The optimal network partition corresponds to that with the minimum value of L [11]. In multilayer networks, the random walk also moves from a node in one layer (e.g. HSPD1 in BRCA) to its counterpart in another layer (e.g. HSPD1 in KIRP) with a given rate called the relax rate. This connects the layers together, allowing Infomap to find modules of chaperones and clients that are connected across cancer types. See details on Infomap and how it is applied to ecological multilayer networks in [12].

Infomap is a fundamentally different approach than SBM. First, it does not require the number of groups to be predetermined. Second, it is not a probabilistic inferential model but rather a descriptive one [14]. Nevertheless, the results of Infomap were nearly identical to those of the SBM. Specifically, we found that the network was clustered to three modules. Two modules had identical chaperone assignments to those of the SBM, and the third module contained SPG7 by itself. Hence, the only difference between the two methods was in the assignment of SPG7. Obtaining almost identical results with two fundamentally different approaches indicates that the signal in the data is extremely strong.

References

1. Shemesh, N. *et al.* The landscape of molecular chaperones across human tissues reveals a layered architecture of core and variable chaperones. *Nat. Commun.* **12,** 2180. doi:10.1038/s41467-021-22369-9 (2021).
2. Paci, P. *et al.* Gene co-expression in the interactome: moving from correlation toward causation via an integrated approach to disease module discovery. *NPJ Syst Biol Appl* **7,** 3. doi:10.1038/s41540-020-00168-0 (2021).
3. Van Dam, S., Võsa, U., van der Graaf, A., Franke, L. & de Magalhães, J. P. Gene co-expression analysis for functional classification and gene-disease predictions. *Brief. Bioinform.* **19,** 575–592. doi:10.1093/bib/bbw139 (2018).
4. Wyrick, J. J. & Young, R. A. Deciphering gene expression regulatory networks. *Curr. Opin. Genet. Dev.* **12,** 130–136. doi:10.1016/s0959-437x(02)00277-0 (2002).
5. Van Waveren, C. & Moraes, C. T. Transcriptional co-expression and co-regulation of genes coding for components of the oxidative phosphorylation system. *BMC Genomics* **9,** 18. doi:10.1186/1471-2164-9-18 (2008).
6. Bie, A. S. *et al.* An inventory of interactors of the human HSP60/HSP10 chaperonin in the mitochondrial matrix space. *Cell Stress Chaperones* **25,** 407–416. doi:10.1007/s12192-020-01080-6 (2020).
7. Ishizawa, J. *et al.* Mitochondrial ClpP-Mediated Proteolysis Induces Selective Cancer Cell Lethality. *Cancer Cell* **35,** 721–737.e9. doi:10.1016/j.ccell.2019.03.014 (2019).
8. Joshi, A. *et al.* The mitochondrial HSP90 paralog TRAP1 forms an OXPHOS-regulated tetramer and is involved in mitochondrial metabolic homeostasis. *BMC Biol.* **18,** 10. doi:10.1186/s12915-020-0740-7 (2020).
9. Chae, Y. C. *et al.* Landscape of the mitochondrial Hsp90 metabolome in tumours. *Nat. Commun.* **4,** 2139. doi:10.1038/ncomms3139 (2013).

10. Szklarczyk, D. *et al.* The STRING database in 2023: protein–protein association networks and functional enrichment analyses for any sequenced genome of interest. *Nucleic Acids Res.* **51,** D638–D646. doi:10.1093/nar/gkac1000 (2022).
11. Rosvall, M. & Bergstrom, C. T. Maps of random walks on complex networks reveal community structure. *Proc. Natl. Acad. Sci. U. S. A.* **105,** 1118–1123. doi:10.1073/pnas.0706851105 (2008).
12. Farage, C., Edler, D., Eklöf, A., Rosvall, M. & Pilosof, S. Identifying flow modules in ecological networks using Infomap. *Methods Ecol. Evol.* **12,** 778–786. doi:10.1111/2041-210x.13569 (2021).
13. De Domenico, M., Lancichinetti, A., Arenas, A. & Rosvall, M. Identifying modular flows on multilayer networks reveals highly overlapping organization in interconnected systems. *Phys. Rev. X* **5,** 011027. doi:10.1103/PhysRevX.5.011027 (2015).
14. Peixoto, T. P. Descriptive vs. inferential community detection in networks: pitfalls, myths, and half-truths (2021).
