# Supplementary material for: Ecological network analysis reveals cancer-dependent chaperone-client interaction structure and robustness
Source: Nat Commun. 2023 Oct 7;14:6277. doi: 10.1038/s41467-023-41906-2 (PMC10560210; doi:10.1038/s41467-023-41906-2)
Supplement: Supplementary file 3 — Reporting Summary [file 41467_2023_41906_MOESM3_ESM.pdf]

nature portfolio

Corresponding author(s): Shai Pilosof
Last updated by author(s): Aug 14, 2023

Reporting Summary

Nature Portfolio wishes to improve the reproducibility of the work that we publish. This form provides structure for consistency and transparency in reporting. For further information on Nature Portfolio policies, see our Editorial Policies and the Editorial Policy Checklist.

Statistics

For all statistical analyses, confirm that the following items are present in the figure legend, table legend, main text, or Methods section.

n/a	Confirmed	
☐	☒	The exact sample size (*n*) for each experimental group/condition, given as a discrete number and unit of measurement
☒	☐	A statement on whether measurements were taken from distinct samples or whether the same sample was measured repeatedly
☐	☒	The statistical test(s) used AND whether they are one- or two-sided<br>*Only common tests should be described solely by name; describe more complex techniques in the Methods section.*
☒	☐	A description of all covariates tested
☒	☐	A description of any assumptions or corrections, such as tests of normality and adjustment for multiple comparisons
☐	☒	A full description of the statistical parameters including central tendency (e.g. means) or other basic estimates (e.g. regression coefficient) AND variation (e.g. standard deviation) or associated estimates of uncertainty (e.g. confidence intervals)
☐	☒	For null hypothesis testing, the test statistic (e.g. *F*, *t*, *r*) with confidence intervals, effect sizes, degrees of freedom and *P* value noted<br>*Give P values as exact values whenever suitable.*
☒	☐	For Bayesian analysis, information on the choice of priors and Markov chain Monte Carlo settings
☒	☐	For hierarchical and complex designs, identification of the appropriate level for tests and full reporting of outcomes
☒	☐	Estimates of effect sizes (e.g. Cohen's *d*, Pearson's *r*), indicating how they were calculated

Our web collection on statistics for biologists contains articles on many of the points above.

Software and code

Policy information about availability of computer code

Data collection	No software was used for data collection.
Data analysis	All analyses were done in R (version 4.1) and Python 2.7 within the Linux environment. Network analysis was done using the packages 'bipartite' (version 2.15) and 'vegan' (version 2.5-7). Algorithm used: Curveball (under 'vegan' package),

For manuscripts utilizing custom algorithms or software that are central to the research but not yet described in published literature, software must be made available to editors and reviewers. We strongly encourage code deposition in a community repository (e.g. GitHub). See the Nature Portfolio guidelines for submitting code & software for further information.

Data

Policy information about availability of data

All manuscripts must include a data availability statement. This statement should provide the following information, where applicable:

- Accession codes, unique identifiers, or web links for publicly available datasets
- A description of any restrictions on data availability
- For clinical datasets or third party data, please ensure that the statement adheres to our policy

Gene level transcriptome profiling (RNA-Seq) data (in the form of HTSeq - FPKM) was download from The Cancer Genome Atlas (TCGA) using the Genomic Data Commons Data Portal https://portal.gdc.cancer.gov). Human protein expression data was downloaded from the string-db.org data base (v11.5) and from published papers. These data and the processed network data are available in figshare (https://doi.org/10.6084/m9.figshare.22779755.v2). The data underlying the figures

and tables are provided in the Source Data file and on Figshare. All code and data are also available on the GitHub repository: Galai G, He X, Rotblat B, Pilosof S. Ecological network analysis reveals cancer-dependent chaperone-client interaction structure and robustness in the mitochondria, authors, title (this paper), https://github.com/Ecological-Complexity-Lab/cancer-networks, DOI: 10.5281/zenodo.8245737, 2023.

Research involving human participants, their data, or biological material

Policy information about studies with human participants or human data. See also policy information about sex, gender (identity/presentation), and sexual orientation and race, ethnicity and racism.

Reporting on sex and gender	N/A
Reporting on race, ethnicity, or other socially relevant groupings	N/A
Population characteristics	N/A
Recruitment	N/A
Ethics oversight	N/A

Note that full information on the approval of the study protocol must also be provided in the manuscript.

Field-specific reporting

Please select the one below that is the best fit for your research. If you are not sure, read the appropriate sections before making your selection.

☒ Life sciences ☐ Behavioural & social sciences ☐ Ecological, evolutionary & environmental sciences

For a reference copy of the document with all sections, see nature.com/documents/nr-reporting-summary-flat.pdf

Life sciences study design

All studies must disclose on these points even when the disclosure is negative.

Sample size	From the Methods section: To ensure a fair comparison between all cancer types we performed a bootstrap analysis to match the sample size of each cancer type to that with the least number of samples (Kidney Renal Papillary Cell Carcinoma, $n=288$). For each cancer type we drew 288 transcriptome samples at random without replacement (1,000 attempts), and reconstructed the chaperone-client network using Spearman correlations (as described above). A correlation that was classified as significant and positive in at least 95\% of the bootstrapping attempts was considered as an interaction.
Data exclusions	No data were excluded
Replication	We reran the code multiple times. Analyses were replicated successfully.
Randomization	Not relevant for this study.
Blinding	Not relevant for this study as this is not an experimental study (there is no human bias while performing the analyses)

Reporting for specific materials, systems and methods

We require information from authors about some types of materials, experimental systems and methods used in many studies. Here, indicate whether each material, system or method listed is relevant to your study. If you are not sure if a list item applies to your research, read the appropriate section before selecting a response.

Materials & experimental systems

n/a	Involved in the study	
☒	☐	Antibodies
☒	☐	Eukaryotic cell lines
☒	☐	Palaeontology and archaeology
☒	☐	Animals and other organisms
☒	☐	Clinical data
☒	☐	Dual use research of concern
☒	☐	Plants

Methods

n/a	Involved in the study	
☒	☐	ChIP-seq
☒	☐	Flow cytometry
☒	☐	MRI-based neuroimaging
